# Bioavailability of Australian pre-schooler iron intakes at specific eating occasions is low

**DOI:** 10.1007/s00394-024-03441-8

**Published:** 2024-06-14

**Authors:** Linda A. Atkins, Sarah A. McNaughton, Alison C. Spence, Lenore J. Evans, Rebecca M. Leech, Ewa A. Szymlek-Gay

**Affiliations:** Institute for Physical Activity and Nutrition (IPAN), School of Exercise and Nutrition Sciences, Deakin University, Melbourne Burwood Campus, 221 Burwood Highway, Burwood, VIC 3125 Australia

**Keywords:** Bioavailability, Iron absorption algorithm, Eating occasion, Heme iron, Pre-schooler, Australia

## Abstract

**Purpose:**

Poor bioavailability may contribute to iron deficiency among children in high-resource countries, but iron bioavailability of Australian pre-schooler diets is unknown. This study aimed to estimate the bioavailability of Australian pre-schooler iron intakes across the day and by eating occasions to identify optimal timing for intervention, by using five previously developed algorithms, and to estimate the proportion of children with intakes of absorbable iron below the requirements.

**Methods:**

Dietary data of children aged 2 to < 6 y (*n* = 812) from the 2011–12 National Nutrition and Physical Activity Survey were collected via two 24-h recalls. Usual food and nutrient intakes were estimated via Multiple Source Method. Phytate, polyphenol, and heme iron values were sourced from international databases or the literature. Five previously published algorithms were applied to observed dietary data to estimate iron bioavailability and calculate the prevalence of children with intakes of absorbable iron below requirements.

**Results:**

Pre-schooler daily iron bioavailability was low (2.7–10.5%) and corresponded to intakes of 0.18–0.75 mg/d of absorbable iron. The proportion of children with inadequate intakes of absorbable iron ranged between 32 and 98%. For all eating occasions, dinner offered iron of the greatest bioavailability (4.2–16.4%), while iron consumed at breakfast was of the lowest bioavailability (1.2–5.6%).

**Conclusion:**

Future strategies are required to improve intakes of bioavailable iron for pre-schoolers to prevent the risk of deficiency. These strategies could include the encouragement of concomitant consumption of enhancers of iron absorption with iron-rich sources, particularly at breakfast.

**Supplementary Information:**

The online version contains supplementary material available at 10.1007/s00394-024-03441-8.

## Introduction

Across the globe, iron deficiency is prevalent, particularly among children under 5 y of age [1, 2]. Optimal iron status is important for immune health, cognitive function, growth and development [3–6], with potentially significant developmental delays resulting from iron deficiency during childhood, some irreversible if iron deficiency is severe [7]. Within high-resource countries, iron deficiency is commonly attributable to inadequate quantity or poor bioavailability of iron [3–9]. Indeed, it is estimated that 12% of young Australian children have inadequate dietary iron intakes [10]. However, among children with seemingly adequate iron intakes, the extent to which these intakes are bioavailable is unknown.

It is well established that bioavailability differs between the two forms of dietary iron, heme and non-heme. Heme iron is more readily absorbed [11], while non-heme iron absorption is affected by dietary modifiers consumed at a similar time, e.g., vitamin C and animal flesh are known enhancers of non-heme iron absorption, while phytates can impose an inhibitory effect [12, 13]. Most (92%) of the iron in the diet of Australian pre-schoolers (children aged 2 to < 6 y) is in the non-heme form [14]. This is not unusual among omnivorous diets in the western world [8, 15], however it may be of concern in terms of bioavailability, and hence the impact on absorption. It is possible for seemingly adequate iron intakes to be insufficiently absorbed. While we previously showed that iron intakes were adequate for more than 80% of Australian pre-schoolers [10], the only pre-schooler dietary pattern to be positively associated with dietary iron intake was characterized by non-heme iron sources, and heme iron intakes were not associated with pre-schooler dietary patterns [14]. Therefore, it is likely that Australian pre-schoolers may absorb insufficient amounts of dietary iron to meet their requirements which are set by modelling requirement components, using estimates for absorbed iron at the 50th centile with upper limits for iron absorption of 14% for 2-3-y-olds, and 18% for 4-5-y-olds [16].

While isotope methods allow for direct measurement of human iron absorption [17, 18], they are often not practical, especially at a population level, as they are costly and time consuming [18]. Iron absorption algorithms are universally accepted as a cost-effective alternative to estimate iron bioavailability for large cohorts [18]. Various algorithms have been developed to estimate dietary iron bioavailability, even in the absence of biochemical data of iron status, by considering iron intake, assumed body iron stores, and the effects of dietary modifiers [19–28]. The application of such algorithms has previously estimated iron bioavailability, which ranged between 1.0% and 8.7%, among toddlers of Kenya, Egypt, and Mexico [22], and children aged 5–10 y of Morocco and India [23, 29]. To date, the existing algorithms have not been applied to the diets of children of high-resource countries, where dietary intakes are likely to differ and sources of readily bioavailable iron may be more accessible. Therefore, the aims of this study were to estimate the bioavailability of Australian pre-schooler iron intakes across the day and eating occasions (EOs) to identify which EO to target for intervention, by using five previously developed algorithms, and to estimate the prevalence of children with intakes of absorbable iron below the requirements.

## Subjects and methods

### Study design and participants

A secondary analysis of data from the National Nutrition and Physical Activity Survey component of the Australian Health Survey was performed for this study, the methodology of which is described elsewhere [30]. Briefly, between May 2011 and June 2012, a sample of 9,519 randomly-selected private dwellings across urban and rural areas of each Australian state and territory was surveyed by trained interviewers from the Australian Bureau of Statistics [30]. A personal interview was conducted with one adult, aged 18 y and over, to provide household demographic and socio-economic data, and one child, aged 2 y and over, to provide individual anthropometric (measured by interviewers) and dietary data (collected with a 24-h recall via an adult proxy for each child). At least eight days later, a second 24-h recall was conducted via telephone (again, via an adult proxy for each child). The total sample comprised 12,153 participants (77% response rate), 822 of which were children aged between 2 and < 6 y. The allocation of separate person weights, which were adjusted for the probability of selection and non-response, allowed for inference to the total Australian population.

The Australian Bureau of Statistics was permitted to conduct the interviews under the Census and Statistics Act 1905. Written, informed consent of adults and legal guardians of children was provided [30].

### Dietary intakes

Dietary data were collected via two 24-h recalls which implemented a version of the United States Department of Agriculture Automated Multiple-Pass Method [31]. The Australian Bureau of Statistics and Food Standards Australia New Zealand modified the Automated Multiple-Pass Method to reflect the Australian food supply [30]. A series of specifically-designed questions prompted respondents to report details of foods and beverages consumed the previous day. Respondent recall of quantities of foods and beverages consumed was assisted by food model booklets which contained images depicting actual sizes of food measures, portions, and containers. The 24-h recall data were coded with the use of the purposely-developed AUSNUT 2011-13 database containing 5,740 foods and nutrient profiles which account for different methods of food preparation [32], and allowed the calculation of energy, nutrients, animal tissue, and tea and coffee intakes.

The calculation of heme and non-heme iron intakes has been described in detail elsewhere [14]. Briefly, a database containing the 2,159 reported food items consumed by 2-5-y-olds was prepared, and foods containing animal tissue (*n* = 529) were identified to facilitate the estimation of heme iron content within these foods, with iron in the remaining foods assumed to be non-heme iron. Details of the type, cut, and preparation method of animal tissue determined the quantity of total iron per 100 g [32]. With reference to analytical data for commonly consumed, cooked, Australian meats [33], the content of heme iron and non-heme iron was calculated for grams of each type of animal tissue. Heme iron proportions for beef, lamb, pork, sausage, liver, poultry, and fish and seafood were 62%, 61%, 65%, 36%, 33%, 62%, 41%, respectively [33]. The disaggregation of mixed dishes containing red meat, poultry, fish, seafood, liver, or sausage was applied for calculation of heme iron content [34–36]. For each child, and for each recall day, intakes of total dietary and heme iron were calculated. Usual intakes were derived via statistical modelling described below. Non-heme iron intakes were calculated for each child, as the difference between total dietary and heme iron intakes.

Food composition values for phytate were necessary for the application of some algorithms, but unavailable within Australian food and nutrient databases. Phytate values were inputted from the New Zealand food data from the EAT-Phytate database [37], where all foods consumed by 2-5-y-old children in the National Nutrition and Physical Activity Survey were matched with equivalent foods in the EAT-Phytate database. Australia and New Zealand share food statutory codes [38], nutrient recommendations [39], and a similar food culture and product availability. When an appropriate New Zealand option was not available to select for mixed foods, recipe data were referred to for grams of individual ingredients [32], for which phytate values were applied, allowing the quantity of phytate consumed to be calculated. Polyphenol values for coffee, and green and black tea were obtained from Phenol-Explorer (version 3.6) [40] and calculated for quantities consumed by participants.

### Eating occasions

For the application of algorithms, the self-reported EO and time of eating information from the 24-h recall were used. During the 24-h recall, respondents were asked to recall the type of EO and the time when that EO commenced. The EO type response options were breakfast, lunch, dinner, supper, brunch, morning tea, afternoon tea, snack, beverage/drink, feeding-infant, extended consumption, and other. The term ‘feeding-infant’ was used to record the consumption of infant or toddler formula, and in some instances, cow’s milk. When the quantity and EO start time were known of foods or beverages that were not consumed within a distinct timeframe, the respondent was able to select ‘extended consumption’. When the available EO options were not suitable, respondents were able to select the ‘other’ category to specify their response.

For this analysis, it was necessary to recode some EO types because of potential ambiguity of terminology, the foods within an EO type, or low frequency of consumption. Breakfast, lunch, and dinner were retained as reported. EOs reported as brunch (0.2% of all EOs) were recoded as breakfast as these mostly occurred near the chronological time of breakfasts and consisted of similar food to breakfasts. Morning and afternoon teas were recoded as snacks. The terms morning and afternoon tea may be used to describe snack foods and drinks or mid-meals provided by child-care facilities, at which attendance in Australia is common (59–72%) for this age group [41]. Suppers were grouped with snacks as they generally consisted of either snack or dessert items and were also of low frequency (0.7%). Beverage/drink, feeding-infant, extended consumption, and other EOs were also recoded as snack in line with previous research [42]. Subsequently, due to the potential effects on non-heme iron bioavailability by similarly timed intake of dietary modifiers, EOs were grouped with the closest occurring EO when reported within one hour from that EO; these grouped EOs retained the name of the main meal EO (e.g., breakfast grouped with a snack eaten within one hour was named ‘breakfast’) [43]. There was no instance where a main meal EO (i.e., breakfast, lunch or dinner) was consumed within one hour from another main meal EO.

The snack group including self-reported and reclassified snack (i.e., morning tea, afternoon tea, beverage/drink, supper, feeding-infant, extended consumption, and other) was then recategorized into early or late, based on the start time of consumption. Early snacks were those consumed prior to midday, while snacks consumed between midday and midnight were termed late snacks.

### Estimation of iron bioavailability

The algorithms of Monsen & Balintfy [20], Murphy et al. [22], Tseng et al. [23], Reddy et al. [25], and Bhargava et al. [26] were applied to observed dietary data for each EO to estimate the bioavailability of pre-schooler iron intakes. These five algorithms were selected based on the availability of variables required and their suitability for the study population (Online Supplementary Table 1). As the recent nationally-representative Australian Health Survey does not include biochemical data of iron status for participants under the age of 12 y [30], only algorithms that do not require this information were considered [20, 22, 23, 25, 26]. All algorithms for which the National Nutrition and Physical Activity Survey provided relevant data were used. For the algorithm of Reddy et al., which estimates bioavailability of non-heme iron only [25], heme iron absorption was assumed to be 25%, as per the algorithm of Murphy et al. [22]. A reference of 500 mg for body iron stores was assumed for the application of the algorithm of Bhargava et al., as the pre-schoolers within our study were not considered to be under-nourished [26]. In applying the algorithm of Tseng et al., the correction by Bhargava et al. was used, where, for intakes below or equal to 2.88 mg phytate, the correction factor was 1. The algorithms of Monsen & Balintfy, Murphy et al., Tseng et al., and Bhargava et al. make an assumption that 40% of total iron in animal tissue is heme iron [20, 22, 23, 26], however we have calculated heme and non-heme iron content of each food as described above. We also used the calculated heme and non-heme iron intakes in the application of the algorithm of Reddy et al. [25].

### Data analysis

Participants aged between 2 and < 6 y, with at least one day of dietary recall data were eligible for this study (*n* = 822). Children were considered misreporters if observed energy intakes were > or < 3SD of the mean for either recall day and these data were excluded (*n* = 10) [14, 44]. A total of 812 participants were included in the final analysis (Online Supplementary Fig. 1). For all analyses, results are presented for two age groups, children aged 2–3 y and 4–5 y, in accordance with the life stages adopted for Australian national nutrient recommendations [39]. All statistical analyses were performed using Stata (Release 16.0; StataCorp LP, College Station, TX, USA).

Demographic and socio-economic characteristics of the children were summarized. Survey person and replicate weights were implemented in these analyses to account for the unequal probabilities of selection and to adjust standard errors (delete-1 jackknife method) for the clustered sample design, respectively.

Both days of dietary data were used to estimate usual daily intakes for energy, nutrients, and factors required by the algorithms for iron bioavailability estimation (e.g., grams of meat, fish, poultry, tea and coffee) for each child via the Multiple Source Method (version 1.0.1, 2011; German Institute of Human Nutrition, Potsdam-Rehbrücke). Publicly available online, Multiple Source Method is a statistical modelling program for estimating usual intakes of nutrients and foods from observed dietary data of individuals and population groups [45, 46].

As two days of dietary data were not available for 324 (39%) individuals, iron bioavailability was estimated for the primary analysis using the first day of dietary recall to ensure independence of observations. Separate estimates were also calculated for the children who provided a second day of dietary data, for supplementary analysis. The bioavailability of iron for each EO, for each child was estimated by inputting the equations of each of the five selected algorithms to the observed dietary data from the first dietary recall into the statistical software Stata. Once the proportion (%) of iron that is bioavailable was estimated for each EO, the amount of absorbable iron (mg) per EO was calculated (i.e., total iron per EO x % bioavailability of iron per EO/100). For each algorithm, these data were summarized as a median percentage iron bioavailability and median (25th, 75th percentile) mg absorbable iron for each EO. The range of iron bioavailability (min % - max %) and amount of absorbable iron (min mg - max mg) are also presented for each EO for all five algorithms. These data were also used to estimate the proportion of iron that is bioavailable (%) and mg absorbable iron (25th, 75th percentile) for each child per day and summarized as group medians for each algorithm; these data are also presented to show the range of daily iron bioavailability (min % - max %) and daily amount of absorbable iron (min mg - max mg) for all five algorithms. The proportion of children below the requirements for absorbed iron (mg) were estimated using the median iron requirements of 0.54 mg/d for 2-3-y-olds and 0.74 mg/d for 4-5-y-olds as cut-off values [16].

## Results

Among the 812 pre-schoolers, 57% were aged 2–3 y, and sex was evenly represented (Table 1). Households predominantly consisted of Australian-born adult respondents, and the majority of adult respondents were employed and tertiary qualified. Usual intakes of selected nutrients and foods are presented in Table 2. Usual mean (SD) total dietary and heme iron intake for 2-3-y-olds was 6.2 (2.0) and 0.5 (0.3) mg/d, respectively, and similar intakes were observed for older pre-schoolers. Usual mean (SD) intakes of animal tissue containing heme iron for younger and older pre-schoolers was 59.7 (22.0) and 60.4 (22.7) g/d, respectively. Dietary iron intake was primarily provided by non-heme iron (92% of total dietary iron) for both age groups. Usual mean (SD) phytate intake was 410.4 (186.4) mg/d for 2-3-y-olds, and 447.2 (166.9) mg/d for 4-5-y-olds, while tea/coffee intakes were low and appeared to increase with age (2.5 to 7.0 mL/d), as did polyphenols from these drinks (1.4 to 3.1 mg/d).

Tables 3 and 4 provide estimated median bioavailability of observed iron intakes of pre-schoolers. The calculated daily (i.e., across all individual EOs across the day per each child) median iron bioavailability ranged between 2.9 and 10.2% for 2-3-y-olds, and 2.7–10.5% for 4-5-y-olds, depending on the algorithm used, which corresponded to the daily median absorbable iron of between 0.18 and 0.70 mg for 2-3-y-olds, and 0.19–0.75 mg for 4-5-y-olds. A large proportion of pre-schoolers (32–98%, depending on the age group and algorithm used) did not consume enough absorbable iron to meet requirements (Table 5).

For all EOs, the greatest median (25th, 75th percentile) observed intakes of iron, were at dinner (1.59 mg (1.00, 2.54 mg)), and breakfast (1.53 mg (0.88, 3.09 mg)), for younger pre-schoolers. For older pre-schoolers, breakfast offered the greatest median (25th, 75th percentile) observed intakes of iron (1.99 mg (1.11, 3.65 mg)), followed by dinner (1.70 mg (1.01, 2.53 mg)). Estimated iron bioavailability for breakfast was lowest for all EOs and ranged between 1.3 and 5.6% for younger, and 1.2–5.6% for older pre-schoolers, depending on the algorithm used. Dinner provided the greatest iron bioavailability for younger (4.7–16.4%) and older (4.2–16.2%) pre-schoolers and offered the greatest amount of absorbable iron (0.06–0.25 mg for both age groups). For all EOs, median (25th, 75th percentile) observed iron intakes were lowest from early snacks, providing 0.52 mg (0.23, 0.99 mg) mg for younger, and 0.49 mg (0.23, 1.05 mg) mg for older pre-schoolers. Although these snacks provided iron of bioavailability similar to other EOs for both younger (2.4 − 10.0%) and older (2.1–10.0%) pre-schoolers, due to their low iron content, they provided the smallest amount of absorbable iron (0.01–0.04 mg for both age groups) for all EOs.

Results for day 2 (Online Supplementary Tables 2 and 3) appeared similar to those of day 1. Estimated daily median iron bioavailability ranged between 2.9 and 10.2% for younger pre-schoolers, and between 2.7 and 10.2% for older pre-schoolers, depending on the algorithm used, with estimated intakes of absorbable iron of 0.19–0.68 mg/d for younger and 0.17–0.71 mg/d for older pre-schoolers. Estimated iron bioavailability, for both age groups, was greatest for dinner (4.6–16.5%), and lowest for breakfast (1.2–5.6%).

## Discussion

To our knowledge, this is the first study to focus on the bioavailability of iron among pre-schooler daily diets and specific EOs in high-resource countries, and to estimate the proportion of children with intakes of absorbable iron below requirements. Using five different previously published algorithms, this study estimated the bioavailability of dietary iron intakes of 812 Australian pre-schoolers to range between 2.7% and 10.5%. The daily median absorbable iron was 0.18–0.75 mg/d. This translates to a very high proportion of pre-schoolers not meeting their requirements for absorbed iron. Although greatest iron intakes were observed at breakfast and dinner, breakfast foods provided the least bioavailable iron and dinner foods provided the highest amount of bioavailable iron. Early snacks, where the lowest iron intakes were observed, provided iron of bioavailability similar to other EOs but the smallest amount of absorbable iron.

High bioavailability of iron at dinner was not surprising given more than half of meals consumed at dinner included animal tissue, which contains highly bioavailable heme iron [11], and most included vegetables, fruit or juice, which are sources of vitamin C, an enhancer of non-heme iron absorption [47]. Early snacks were composed of mostly milk, yoghurt, fruit, juice, and discretionary foods, such as cakes and biscuits. It is likely that the vitamin C provided by fruit and juice enhanced iron bioavailability within these foods. Vitamin C can improve iron absorption considerably [48–50], and counteract the inhibitory effects of phytates and polyphenols [51]. In fact, vitamin C’s enhancing effect on iron absorption is said to be even more pronounced in the presence of phytates or polyphenols [47]. While polyphenol content was low, phytate was present in the discretionary foods consumed for early snacks. Breakfast foods were predominantly dairy milk, breakfast cereals, breads, and spreads. These breads and breakfast cereals (some iron-fortified) also contain phytate, but as vitamin C-rich foods were not prominent at this EO, iron absorption was not optimized. Of the 1.53–1.99 mg iron consumed at breakfast across both age groups, as little as 0.02 and no more than 0.12 mg was likely to be absorbable.

We applied five algorithms [20, 22, 23, 25, 26], all of which varied in their consideration of factors known to influence non-heme iron absorption, as well as percentages applied for heme iron absorption, which for most was either 23% [20, 23] or 25% [22]. Common to all the algorithms used was the application of factors to account for the enhancing effects of vitamin C and animal tissue [20, 22, 23, 25, 26].

Our results are not directly comparable to those in the literature as most estimate iron bioavailability with reference to biochemical data of iron status [47, 52–54] or have been based on adult or vegetarian populations [18, 21, 24, 27, 28, 52, 54], or diets within low-income nations [18, 22, 23, 26, 29, 54]. When Rani et al. [18] applied the algorithm of Monsen and Balintfy [20] to the vegetarian diet of Indian children (aged 5–8 y), iron bioavailability was 3.5%, which was lower than we observed using this algorithm among 2-5-y-old children (5.4–5.5%), and not surprising given the factor they used for animal tissue was zero. Although small quantities were consumed by Australian pre-schoolers, the presence of some animal tissue in the diet most likely explains the difference in results.

The algorithms of Tseng et al. [23], and Reddy et al. [25], were applied to the predominantly cereal- and legume-based diets of a small sample of 6-10-y-old Moroccan children [29]. The algorithm of Reddy et al. [25], estimated iron bioavailability of Moroccan children to range between 0.3% and 1.0% [29], which is much lower than what was estimated in the current study (8.1–8.3%). The algorithm of Reddy et al. includes a factor for the inhibiting effect of phytate, a rich element of the diet within low-resource countries [25]. Phytate intake among Moroccan children was almost four times greater than for Australian pre-schoolers observed in our study [29]. Additionally, consumption of animal tissue and vitamin C was lower among Moroccan children [29], compared to intakes reported in the current study.

While the algorithm of Tseng et al. [23] estimated an iron bioavailability of 4.3% for Moroccan children’s diets that was more similar to our findings (i.e., 3.0–3.2%), this value was derived for children with low iron stores [29]. The algorithm of Tseng et al. was developed for adults and children with high phytate and tea consumption [23]. Using their algorithm, Tseng et al. reported iron bioavailability of up to 10.8% for Russian children aged 0–6 y [23].

The lowest estimates for iron bioavailability of pre-schooler diets were produced by the algorithm of Bhargava et al. [26], which was developed for adult diets with high phytate and low tea intakes. The factors in this algorithm may underestimate the effects of enhancing and overestimate the effects of inhibitory foods for our study population.

The highest results for possible dietary iron bioavailability for pre-schoolers were produced by the algorithm of Murphy et al. which was developed especially for young children in countries where tea was common among the predominantly cereal-based diets. Tea was not frequently consumed by Australian pre-schoolers (< 5%), but phytate was.

The current Australian/New Zealand recommended intake for iron for 2-3-y-olds is based on the assumption that 14% of iron in the diet is absorbed [39]. For the younger pre-schoolers in this study, the algorithms we applied estimated iron bioavailability for their diets to be only as high as 10.2%, and as low as 2.9% [20, 22, 23, 25, 26]. For children aged 4–5 y, current Australian/New Zealand recommended intakes assume 18% absorption [39]. Our results indicate an estimated 2.7 − 10.5% bioavailability [20, 22, 23, 25, 26]. While the algorithms we applied showed a large range of iron bioavailability, it is clear that very little iron may be absorbed by Australian pre-schoolers (0.18–0.70 mg/d, or 0.19–0.75 mg/d) as most algorithms estimated that dietary iron intake may be insufficiently absorbed for 32–98% of children. Confirmation of these estimates requires biochemical data, which was not collected. Previous studies that have assessed status indicate that prevalence of iron deficiency among Australian children < 6 years ranges between 2 and 25% [55–59].

Future strategies are required to improve intakes of bioavailable iron for pre-schoolers to prevent the risk of deficiency. Our research suggests these strategies could include the encouragement of concomitant consumption of enhancers of iron absorption (e.g., vitamin C-rich foods such as fruit or vegetables) with iron-rich sources (e.g., lean meat, legumes, or whole-grains), particularly at breakfast. Pre-schooler breakfasts currently comprise very little meat, which is mostly consumed at dinner [60]. Strategies to include more iron at early snacks should also be considered. For example, discretionary foods, such as cakes and biscuits, which were commonly consumed by pre-schoolers, could be replaced with nutritious options, such as fruit and sandwiches. Milk beverages could be limited to allow for iron-rich-snacks to be included.

Strengths of this study include the use of the most recent, comprehensive, nationally-representative dietary recall data [30]. Although an appropriate algorithm has not been established for the current study population, this study is further strengthened by the application of all possible algorithms, each offering alternative approaches and considerations in the estimation of iron bioavailability of Australian pre-schooler diets and specific EOs, which allowed for the assessment of the effect of enhancers and inhibitors consumed at the same time as iron intakes. Limitations of this study should also be considered. Currently, there are no Australian food composition data on phytates, polyphenols, heme and non-heme iron content, so these values were sourced from the literature. Blood samples were not collected in this study, therefore we were limited in our selection of algorithms and could not assess the efficiency of the estimated absorption values.

In summary, low iron bioavailability indicated intakes of 0.18–0.75 mg/d of absorbable iron for Australian pre-schoolers, with most children consuming inadequate amounts of absorbable iron. For all EOs, dinner offered iron of the greatest bioavailability and iron consumed at breakfast was of the lowest bioavailability. Strategies are required to increase heme iron intake, where possible, and enhance non-heme iron absorption.

**Table 1:** Characteristics of pre-schoolers from the 2011 – 12 Australian National Nutrition and Physical Activity Survey (*n* = 812)

	2–3 y		4–5 y	
	*n*	%	*n*	%
Sex				
male	225	49	173	49
female	235	51	179	51
BMI category^a^				
underweight	18	4	13	4
normal range	241	52	222	63
overweight	99	22	52	15
missing	102	22	65	18
Country of birth of adult respondent				
Australia	320	70	250	71
main English speaking countries^b^	39	8	27	8
other countries	83	18	63	18
missing	18	4	12	3
Level of education of adult respondent				
low (up to completion of final year of secondary school)	117	25	89	25
medium (trade certificate or diploma)	157	34	137	39
high (undergraduate university degree or higher)	162	35	111	32
missing	24	5	15	4
Employment status of adult respondent				
employed	309	67	243	69
unemployed/not in work force	133	29	97	28
missing	18	4	12	3
Index of relative socio-economic disadvantage of household^c^				
lowest quintile	81	18	68	19
second quintile	89	19	66	19
third quintile	97	21	66	19
fourth quintile	85	19	67	19
highest quintile	108	23	85	24
Food security of household				
ran out of food in last 12 months	35	8	17	5
did not run out of food in last 12 months	410	89	324	92
missing	15	3	11	3
Household type				
two-parent family	370	80	270	77
single-parent family	47	10	53	15
other households	43	9	29	8
Household size				
2 persons per household	22	5	19	5
3 persons per household	136	30	68	19
4 persons per household	199	43	171	49
5 persons per household	84	18	70	20
6 or more persons per household	19	4	24	7

y: years; BMI: body mass index

^a^ BMI categories are based on the following half-year cut off points for individual age and sex. Males; 2 y: < 15.14 underweight, 15.14–18.41 normal, > 18.41 overweight; 2.5 y: < 14.92 underweight, 14.92–18.13 normal, > 18.13 overweight; 3 y: < 14.74 underweight, 14.74–17.89 normal, > 17.89 overweight; 3.5 y: < 14.47 underweight, 14.47–17.69 normal, > 17.69 overweight; 4 y: < 14.43 underweight, 14.43–17.55 normal, > 17.55 overweight; 4.5 y: < 14.31 underweight, 14.31–17.47 normal, > 17.47 overweight; 5 y: < 14.21 underweight, 14.21–17.42 normal, > 17.42 overweight; 5.5 y: < 14.13 underweight, 14.13–17.45 normal, > 17.45 overweight. Females; 2 y: < 14.83 underweight, 14.83–18.02 normal, > 18.02 overweight; 2.5 y: < 14.63 underweight, 14.63–17.76 normal, > 17.76 overweight; 3 y: < 14.47 underweight, 14.47–17.56 normal, > 17.56 overweight; 3.5 y: < 14.32 underweight, 14.32–17.40 normal, > 17.40 overweight; 4 y: < 14.19 underweight, 14.19–17.28 normal, > 17.28 overweight; 4.5 y: < 14.06 underweight, 14.06–17.19 normal, > 17.19 overweight; 5 y: < 13.94 underweight, 13.94–17.15 normal, > 17.15 overweight; 5.5 y: < 13.86 underweight, 13.86–17.20 normal, > 17.20 overweight [30, 61, 62]

^b^ Canada, Ireland, New Zealand, South Africa, United Kingdom, United States of America

^c^ Socio-Economic Indexes for Areas (SEIFA) 2011

**Table 2:** Daily usual dietary intakes^a^ of pre-schoolers from the 2011–12 Australian National Nutrition and Physical Activity Survey

	2–3 y (n = 460)			4–5 y (n = 352)		
	Mean	SD	95%CI	Mean	SD	95%CI
Energy (kJ)	4781	969	4670, 4891	5074	1007	4929, 5219
Fat (g)	39.8	9.6	38.6, 40.9	42.2	11.1	40.7, 43.7
Carbohydrate (g)	144.6	32.4	140.8, 148.5	154.9	31.5	149.4, 160.3
Protein (g)	46.4	11.4	45.0, 47.7	47.9	11.8	46.2, 49.5
Fiber (g)	12.9	4.1	12.4, 13.4	13.8	4.0	13.1, 14.5
Calcium (mg)	623.4	193.6	601.2, 645.6	596.5	203.5	569.7, 623.3
Phytate (mg)	410.4	186.4	385.2, 435.6	447.2	166.9	421.5, 472.9
Tea/coffee (mL)	2.5	17.4	0.1, 4.9	7.0	39.3	1.0, 13.1
Polyphenols from tea/coffee (mg)	1.4	11.1	0.4, 3.2	3.1	21.1	0.2, 6.4
Vitamin C (mg)	79.3	55.5	72.9, 85.8	101.9	129.0	81.7, 122.1
Meat/fish/poultry (g)	59.7	22.0	56.6, 62.7	60.4	22.7	57.7, 63.1
Iron (mg)						
total (dietary plus supplemental)	6.4	2.1	6.2, 6.7	6.6	1.8	6.3, 6.8
dietary	6.2	2.0	6.0, 6.4	6.3	1.7	6.1, 6.6
heme	0.5	0.3	0.5, 0.6	0.5	0.3	0.5, 0.5
non-heme	5.7	2.0	5.5, 5.9	5.8	1.6	5.6, 6.1

y: years

^a^Generated with the Multiple Source Method [63]

**Table 3:** Estimated bioavailability of dietary iron consumed across the day and at specific EOs by 2-3-y-olds from the 2011–12 Australian National Nutrition and Physical Activity Survey shown as the proportion of consumed iron that is bioavailable (median %) and amount that is absorbable (median (25th, 75th percentile) mg)

	Median observed iron intake	Median bioavailable iron											
		Monsen & Balintfy 1982 [20]		Murphy et al.1992 [22]		Tseng et al. 1997 [23]		Reddy et al. 2000 [25]		Bhargava et al. 2001 [26]		Range	
	mg (25th, 75th percentile)	**%**	mg (25th, 75th percentile)	**%**	mg (25th, 75th percentile)	**%**	mg (25th, 75th percentile)	**%**	mg (25th, 75th percentile)	**%**	mg (25th, 75th percentile)	**%**	mg
Median daily intake and bioavailability of iron consumed by children (n = 460)	6.87 (4.66, 9.30)	**5.4**	0.39 (0.26, 0.57)	**10.2**	0.70 (0.47, 0.98)	**3.2**	0.20 (0.13, 0.31)	**8.3**	0.51 (0.34, 0.81)	**2.9**	0.18 (0.12, 0.27)	**2.9–10.2**	0.18–0.70
Breakfast (n = 453)	1.53 (0.88, 3.09)	**3.1**	0.06 (0.03, 0.12)	**5.0**	0.12 (0.05, 0.21)	**1.3**	0.02 (0.01, 0.04)	**5.6**	0.08 (0.04, 0.14)	**1.3**	0.02 (0.01, 0.04)	**1.3–5.6**	0.02–0.12
Lunch (n = 419)	1.20 (0.79, 1.79)	**5.2**	0.06 (0.04, 0.12)	**15.0**	0.18 (0.06, 0.23)	**2.5**	0.03 (0.01, 0.06)	**7.0**	0.07 (0.04, 0.14)	**2.3**	0.03 (0.01, 0.05)	**2.3–15.0**	0.03–0.18
Dinner (n = 443)	1.59 (1.00, 2.54)	**8.9**	0.13 (0.07, 0.25)	**16.4**	0.24 (0.13, 0.40)	**5.8**	0.08 (0.04, 0.16)	**12.3**	0.17 (0.08, 0.38)	**4.7**	0.07 (0.03, 0.13)	**4.7–16.4**	0.07–0.24
Early snacks (n = 384)	0.52 (0.23, 0.99)	**3.5**	0.02 (0.01, 0.04)	**10.0**	0.04 (0.02, 0.08)	**2.4**	0.01 (0.01, 0.02)	**7.2**	0.03 (0.02, 0.06)	**2.4**	0.01 (0.01, 0.02)	**2.4–10.0**	0.01–0.04
Late snacks (n = 434)	0.70 (0.33, 1.31)	**3.4**	0.03 (0.01, 0.06)	**5.0**	0.05 (0.02, 0.11)	**2.0**	0.01 (0.01, 0.03)	**6.9**	0.04 (0.02, 0.08)	**2.0**	0.01 (0.01, 0.03)	**2.0–6.9**	0.01–0.05

EO: eating occasion

Percentages are bold font for readability. Early snacks are snacks consumed after midnight and before midday. Late snacks are snacks consumed between midday and midnight.

**Table 4:** Estimated bioavailability of dietary iron consumed across the day and at specific EOs by 4-5-y-olds from the 2011–12 Australian National Nutrition and Physical Activity Survey shown as the proportion of consumed iron that is bioavailable (median %) and amount that is absorbable (median (25th, 75th percentile) mg)

	Median observed iron intake	Median bioavailable iron															
		Monsen & Balintfy 1982 [20]			Murphy et al.1992 [22]		Tseng et al. 1997 [23]			Reddy et al. 2000 [25]			Bhargava et al. 2001 [26]			Range	
	mg (25th, 75th percentile)	%	mg (25th, 75th percentile)	%		mg (25th, 75th percentile)	%	mg (25th, 75th percentile)	%		mg (25_th_, 75th percentile)	%		mg (25_th_, 75th percentile)		%	mg
Median daily intake and bioavailability of iron consumed by children (*n* = 352)	7.47 (5.42, 9.98)	**5.5**	0.42 (0.30, 0.61)	**10.5**		0.75 (0.55, 1.07)	**3.0**	0.21 (0.14, 0.31)	**8.1**		0.57 (0.37, 0.87)	**2.7**		0.19 (0.13, 0.28)		**2.7–10.5**	0.19–0.75
Breakfast (*n* = 346)	1.99 (1.11, 3.65)	**3.2**	0.07 (0.04, 0.14)	**5.0**		0.12 (0.06, 0.25)	**1.2**	0.03 (0.01, 0.05)	**5.6**		0.09 (0.05, 0.17)	**1.2**		0.03 (0.01, 0.05)		**1.2–5.6**	0.03–0.12
Lunch (*n* = 340)	1.51 (1.03, 2.10)	**5.3**	0.08 (0.04, 0.15)	**15.0**		0.15 (0.08, 0.27)	**2.2**	0.03 (0.01, 0.07)	**6.5**		0.09 (0.05, 0.16)	**2.1**		0.03 (0.01, 0.06)		**2.1–15.0**	0.03–0.15
Dinner (*n* = 347)	1.70 (1.01, 2.53)	**8.7**	0.14 (0.07, 0.23)	**16.2**		0.25 (0.12, 0.41)	**5.0**	0.08 (0.03, 0.14)	**11.8**		0.19 (0.09, 0.36)	**4.2**		0.06 (0.03, 0.12)		**4.2–16.2**	0.06–0.25
Early snacks (*n* = 281)	0.49 (0.23, 1.05)	**3.6**	0.02 (0.01, 0.05)	**10.0**		0.04 (0.02, 0.09)	**2.1**	0.01 (0.00, 0.02)	**7.0**		0.03 (0.02, 0.07)	**2.1**		0.01 (0.00, 0.02)		**2.1–10.0**	0.01–0.04
Late snacks (*n* = 330)	0.75 (0.36, 1.53)	**3.6**	0.03 (0.01, 0.08)	**5.0**		0.06 (0.03, 0.13)	**2.0**	0.01 (0.01, 0.03)	**6.8**		0.04 (0.02, 0.09)	**2.0**		0.01 (0.01, 0.03)		**2.0–6.8**	0.01–0.06

EO: eating occasion

Percentages are bold font for readability. Early snacks are snacks consumed after midnight and before midday. Late snacks are snacks consumed between midday and midnight.

**Table 5:** Proportion of children from the 2011–12 Australian National Nutrition and Physical Activity Survey with intakes of absorbable iron below requirements^a^

Algorithm	2–3 y (*n* = 460) %	4–5 y (*n* = 352) %
Monsen & Balintfy 1982 [20]	73	87
Murphy et al.1992 [22]	32	48
Tseng et al. 1997 [23]	91	97
Reddy et al. 2000 [25]	53	67
Bhargava et al. 2001 [26]	95	98

^a^ The median requirement for absorbed iron for 2-3-y-olds is 0.54 mg/d and for 4-5-y-olds is 0.74 mg/d [16]

## Electronic supplementary material

Below is the link to the electronic supplementary material.

Supplementary Material 1

Supplementary Material 2

## Data Availability

Data described in the manuscript, code book, and analytic code may be made available upon request pending application to and approval by the Australian Bureau of Statistics, and payment of any necessary data fees: https://www.abs.gov.au/statistics/microdata-tablebuilder/available-microdata-tablebuilder/australian-health-survey-nutrition-and-physical-activity.

## Declarations

### Disclaimers

Nil.

### Authors? last names for PubMed indexing

Atkins, McNaughton, Spence, Evans, Leech, Szymlek-Gay.

## References

[1] McLean E, Cogswell M, Egli I, Wojdyla D, de Benoist B (2009) Worldwide prevalence of anaemia, WHO Vitamin and Mineral Nutrition Information System, 1993–2005. Public Health Nutr 12(04):444–454. 10.1017/S1368980008002401 18498676 10.1017/S1368980008002401

[2] World Health Organization, United Nations Children’s Fund, United Nations University (2001) Iron Deficiency Anaemia: Assessment, Prevention and Control. A guide for Programme Managers. World Health Organization. http://whqlibdoc.who.int/hq-/2001./WHO_NHD_01.3.pdf Accessed 26 May 2017

[3] Georgieff MK (2007) Nutrition and the developing brain: nutrient priorities and measurement. Am J Clin Nutr 85(2):614S–620S 17284765 10.1093/ajcn/85.2.614S

[4] Rao R, Georgieff MK (2007) Iron in fetal and neonatal nutrition. Seminars Fetal Neonatal Med 12(1):54–63. 10.1016/j.siny.2006.10.007 10.1016/j.siny.2006.10.007 PMC2048487 17157088

[5] Beard JL (2008) Why Iron Deficiency is important in Infant Development. J Nutr 138(12):2534–2536 19022985 10.1093/jn/138.12.2534 PMC3415871

[6] Grantham-McGregor S, Ani C (2001) A review of studies on the effect of iron deficiency on cognitive development in children. J Nutr 131(2):649S–668S 11160596 10.1093/jn/131.2.649S

[7] Lozoff B, Beard J, Connor J, Felt B, Georgieff M, Schallert T (2006) Long-lasting neural and behavioral effects of iron deficiency in infancy. Nutr Rev 64(5 Part 2):S34–43 16770951 10.1301/nr.2006.may.S34-S43 PMC1540447

[8] Thane CW, Walmsley CM, Bates CJ, Prentice A, Cole TJ (2000) Risk factors for poor iron status in British toddlers: further analysis of data from the national diet and nutrition survey of children aged 1.5–4.5 years. Public Health Nutr 3(4):433–440 11135798 10.1017/s1368980000000501

[9] Atkins LA, Spence AC, Szymlek-Gay EA (2023) Iron Nutrition of pre-schoolers in High-Income countries: a review. Nutrients 15(11). 10.3390/nu15112616 10.3390/nu15112616 PMC10255056 37299582

[10] Atkins LA, McNaughton SA, Spence AC, Szymlek-Gay EA (2020) Adequacy of iron intakes and socio-demographic factors associated with iron intakes of Australian pre-schoolers. Eur J Nutr 59(1):175–184. 10.1007/s00394-019-01897-7 30707362 10.1007/s00394-019-01897-7

[11] Hallberg L, Hoppe M, Andersson M, Hulthén L (2003) The role of meat to improve the critical iron balance during weaning. Pediatrics 111(4):864–870 12671125 10.1542/peds.111.4.864

[12] Cook JD, Layrisse M, Martinez-Torres C, Walker R, Monsen E, Finch CA (1972) Food Iron absorption measured by an extrinsic tag. J Clin Invest 51(4):805–815. 10.1172/JCI106875 5062612 10.1172/JCI106875 PMC302194

[13] Layrisse M, Martínez-Torres C, Roche M (1968) Effect of interaction of various foods on iron absorption. Am J Clin Nutr 21(10):1175–1183 5686245 10.1093/ajcn/21.10.1175

[14] Atkins LA, McNaughton SA, Spence AC, Szymlek-Gay EA (2021) Dietary patterns of Australian pre-schoolers and associations with haem and non-haem iron intakes. Eur J Nutr 60(6):3059–3070. 10.1007/s00394-020-02477-w 33484317 10.1007/s00394-020-02477-w

[15] Rickard AP, Chatfield MD, Conway RE, Stephen AM, Powell JJ (2009) An algorithm to assess intestinal iron availability for use in dietary surveys. Br J Nutr 102(11):1678–1685. 10.1017/S0007114509990894 19709447 10.1017/S0007114509990894 PMC3065058

[16] Institute of Medicine, Food and Nutrition Board (2001) Dietary Reference Intakes for Vitamin A, Vitamin K, Arsenic, Boron, Chromium, Copper, Iodine, Iron, Manganese, Molybdenum, Nickel, Silicon, Vanadium, and Zinc. The National Academies Press. http://www.nap.edu/openbook.php?record_id=10026. Accessed 26 May 2017 25057538

[17] Fomon SJ, Nelson SE, Serfass RE, Ziegler EE (2005) Absorption and loss of Iron in toddlers are highly correlated. J Nutr 135(4):771–777 15795433 10.1093/jn/135.4.771

[18] Rani V, Trijsburg L, Brouwer ID, Khetarpaul N (2010) Dietary non-heme iron bioavailability among children (ages 5–8) in a rural, high-anemia-prevalent area in North India: comparison of algorithms. Ecol Food Nutr 49(4):262–278 21888471 10.1080/03670244.2010.491050

[19] Monsen ER, Hallberg L, Layrisse M, Hegsted DM, Cook JD, Mertz W, Finch CA (1978) Estimation of available dietary iron. Am J Clin Nutr 31(1):134–141 619599 10.1093/ajcn/31.1.134

[20] Monsen ER, Balintfy JL (1982) Calculating dietary iron bioavailability: refinement and computerization. J Am Diet Assoc 80(4):307–311 7061776

[21] Cook JD, Dassenko SA, Lynch SR (1991) Assessment of the role of nonheme-iron availability in iron balance. Am J Clin Nutr 54(4):717–722 1897479 10.1093/ajcn/54.4.717

[22] Murphy SP, Beaton GH, Calloway DH (1992) Estimated mineral intakes of toddlers: predicted prevalence of inadequacy in village populations in Egypt, Kenya, and Mexico. Am J Clin Nutr 56(3):565–572 1503070 10.1093/ajcn/56.3.565

[23] Tseng M, Chakraborty H, Robinson DT, Mendez M, Kohlmeier L (1997) Adjustment of Iron Intake for Dietary enhancers and inhibitors in Population studies: Bioavailable Iron in Rural and Urban Residing Russian Women and Children. J Nutr 127(8):1456–1468 9237938 10.1093/jn/127.8.1456

[24] Du S, Zhai F, Wang Y, Popkin BM (2000) Current methods for estimating Dietary Iron Bioavailability do not work in China. J Nutr 130(2):193–198 10720169 10.1093/jn/130.2.193

[25] Reddy MB, Hurrell RF, Cook JD (2000) Estimation of nonheme-iron bioavailability from meal composition. Am J Clin Nutr 71(4):937–943 10731500 10.1093/ajcn/71.4.937

[26] Bhargava A, Bouis HE, Scrimshaw NS (2001) Dietary intakes and Socioeconomic Factors Are Associated with the Hemoglobin concentration of Bangladeshi women. J Nutr 131(3):758–764 11238756 10.1093/jn/131.3.758

[27] Chiplonkar S, Agte V (2006) Statistical model for predicting non-heme iron bioavailability from vegetarian meals. Int J Food Sci Nutr 57(7–8):434–450 17162323 10.1080/09637480600836833

[28] Conway RE, Powell JJ, Geissler CA (2007) A food-group based algorithm to predict non-heme iron absorption. Int J Food Sci Nutr 58(3):29–41 17415954 10.1080/09637480601121250

[29] Zimmermann MB, Chaouki N, Hurrell RF (2005) Iron deficiency due to consumption of a habitual diet low in bioavailable iron: a longitudinal cohort study in Moroccan children. Am J Clin Nutr 81:115–121 15640469 10.1093/ajcn/81.1.115

[30] Australian Bureau of Statistics (2013) Australian Health Survey: Users’ Guide, 2011-13, cat. no. 4363.0.55.001. ABS. http://www.abs.gov.au/ausstats/abs@.nsf/Lookup/74D87E30B3539C53CA257BBB0014BB36?opendocument Accessed 26 May 2017

[31] Blanton CA, Moshfegh AJ, Baer DJ, Kretsch MJ (2006) The USDA Automated multiple-pass method accurately estimates group total energy and nutrient intake. J Nutr 136(10):2594–2599 16988132 10.1093/jn/136.10.2594

[32] Food Standards Australia New Zealand (2015) AUSNUT 2011-13- Australian Food, Supplement and Nutrient Database for Estimation of Population Nutrient Intakes. FSANZ. http://www.foodstandards.gov.au/science/monitoringnutrients/ausnut/Pages/about.aspx. Accessed 16 April 2018

[33] Rangan AM, Ho RWL, Blight GD, Binns CW (1997) Haem iron content of Australian meats and fish. Food Aust 49(11):508–511

[34] Cosgrove M, Flynn A, Kiely M (2005) Impact of disaggregation of composite foods on estimates of intakes of meat and meat products in Irish adults. Public Health Nutr 8(3):327–337. 10.1079/PHN2004692 15918931 10.1079/phn2004692

[35] Prynne CJ, Wagemakers JJMF, Stephen AM, Wadsworth MEJ (2009) Meat consumption after disaggregation of meat dishes in a cohort of British adults in 1989 and 1999 in relation to diet quality. Eur J Clin Nutr 63(5):660–666. 10.1038/ejcn.2008.7 18285805 10.1038/ejcn.2008.7 PMC2766765

[36] Sui Z, Raubenheimer D, Rangan A (2017) Consumption patterns of meat, poultry, and fish after disaggregation of mixed dishes: secondary analysis of the Australian National Nutrition and Physical Activity Survey 2011–12. BMC Nutr 3(1):1–12. 10.1186/s40795-017-0171-1 10.1186/s40795-017-0171-1 PMC7050704 32153832

[37] Hartley NK (2014) Phytate and Zinc intakes of New Zealand toddlers aged 12–24 months. University of Otago, Dunedin

[38] Food Standards Australia New Zealand (2012) Australia New Zealand Food Standards Code. FSANZ. http://www.foodstandards.gov.au/foodstandards/foodstandardscode.cfm. Accessed 19 May 2012

[39] National Health and Medical Research Council (2006) Nutrient Reference Values for Australia and New Zealand Including Recommended Dietary Intakes. Commonwealth of Australia. https://www.nhmrc.gov.au/guidelines-publications/n35-n36-n37. Accessed 26 May 2017

[40] Rothwell JA, Perez-Jimenez J, Neveu V, Medina-Remón A, M’Hiri N, García-Lobato P, Manach C, Knox C, Eisner R, Wishart DS, Scalbert A (2013) Phenol-Explorer 3.0: a major update of the Phenol-Explorer database to incorporate data on the effects of food processing on polyphenol content. Database 2013. 10.1093/database/bat070 10.1093/database/bat070 PMC3792339 24103452

[41] Childhood Education and Care, Australian Bureau of Statistics, Australia J (2018) 2017, Data Cube 4402.0. https://www.abs.gov.au/AUSSTATS/abs@.nsf/DetailsPage/4402.0June%202017?OpenDocument

[42] Leech RM, Spence AC, Lacy KE, Zheng M, Timperio A, McNaughton SA (2021) Characterizing children’s eating patterns: does the choice of eating occasion definition matter? Int J Behav Nutr Phys Act 18(1):165. 10.1186/s12966-021-01231-7 34923993 10.1186/s12966-021-01231-7 PMC8684678

[43] Fuzi SFA, Koller D, Bruggraber S, Pereira DIA, Dainty JR, Mushtaq S (2017) A 1-h time interval between a meal containing iron and consumption of tea attenuates the inhibitory effects on iron absorption: a controlled trial in a cohort of healthy UK women using a stable iron isotope. Am J Clin Nutr 106(6):1413–1421. 10.3945/ajcn.117.161364 29046302 10.3945/ajcn.117.161364

[44] Atkins LA, McNaughton SA, Campbell KJ, Szymlek-Gay EA (2016) Iron intakes of Australian infants and toddlers: findings from the Melbourne Infant Feeding, Activity and Nutrition Trial (InFANT) program. Br J Nutr 115(02):285–293. 10.1017/S0007114515004286 26571345 10.1017/S0007114515004286

[45] Lei L, Rangan AM, Flood VM, Louie JCY (2016) Dietary intake and food sources of added sugar in the Australian population – CORRIGENDUM...Lei L, Rangan A, Flood VM, et al. (2016) Dietary intake and food sources of added sugar in the Australian population. British Journal of Nutrition 115, 868–877. 116:1136–1136. 10.1017/S0007114516003093 10.1017/S0007114515005255 26794833

[46] Livingstone KM, McNaughton SA (2017) Dietary patterns by reduced rank regression are associated with obesity and hypertension in Australian adults 10.1017/S0007114516004505 28120736

[47] Hallberg L, Hulthén L (2000) Prediction of dietary iron absorption: an algorithm for calculating absorption and bioavailability of dietary iron [corrected] [published erratum appears in AM J CLIN NUTR 2000; 72(5): 1242]. Am J Clin Nutr 71(5):1147–1160 10799377 10.1093/ajcn/71.5.1147

[48] Food and Agriculture Organization, World Health Organization (2002) Human vitamin and mineral requirements. Report of a joint FAO/WHO expert consultation Bangkok, Thailand., Rome

[49] Hallberg L, Brune M, Rossander L (1986) Effect of ascorbic-acid on iron-absorption from different types of meals - studies with ascorbic-acid-rich foods and synthetic ascorbic-acid given in different amounts with different meals. Hum Nutrition-Applied Nutr 40A(2):97–113 3700141

[50] Hurrell RF, Reddy MB, Juillerat M, Cook JD (2003) Degradation of phytic acid in cereal porridges improves iron absorption by human subjects. Am J Clin Nutr 77(5):1213–1219 12716674 10.1093/ajcn/77.5.1213

[51] Hallberg L, Brune M, Rossander L (1989) Iron absorption in man: ascorbic acid and dose-dependent inhibition by phytate. Am J Clin Nutr 49(1):140–144 2911999 10.1093/ajcn/49.1.140

[52] Dainty JR, Berry R, Lynch SR, Harvey LJ, Fairweather-Tait SJ (2014) Estimation of Dietary Iron Bioavailability from Food Iron Intake and Iron Status. PLoS ONE 9(10):1 10.1371/journal.pone.0111824 PMC4214798 25356629

[53] Armah SM, Carriquiry A, Sullivan D, Cook JD, Reddy MB (2013) A complete diet-based algorithm for predicting nonheme iron absorption in adults. J Nutr 143(7):1136–1140. 10.3945/jn.112.169904 23700342 10.3945/jn.112.169904

[54] Beard JL, Murray-Kolb LE, Haas JD, Lawrence F (2007) Iron absorption prediction equations lack agreement and Underestimate Iron absorption. J Nutr 137(7):1741–1746 17585024 10.1093/jn/137.7.1741

[55] Zhou SJ, Gibson RA, Gibson RS, Makrides M (2012) Nutrient intakes and status of preschool children in Adelaide, South Australia. Med J Aust 196(11):696–700 22708768 10.5694/mja11.11080

[56] Karr M, Alperstein G, Causer J, Mira M, Lammi A, Fett MJ (1996) Iron status and anaemia in preschool children in Sydney. Aust N Z J Public Health 20(6):618–622. 10.1111/j.1467-842X.1996.tb01076.x 9117969 10.1111/j.1467-842x.1996.tb01076.x

[57] Karr MA, Mira M, Alperstein G, Labib S, Webster BH, Lammi AT, Beal P (2001) Iron deficiency in Australian-born children of arabic background in central Sydney. Med J Aust 174(4):165–168 11270755 10.5694/j.1326-5377.2001.tb143208.x

[58] Oti-Boateng P, Seshadri R, Petrick S, Gibson RA, Simmer K (1998) Iron status and dietary iron intake of 6-24-month-old children in Adelaide. J Paediatr Child Health 34(3):250–253 9633972 10.1046/j.1440-1754.1998.00205.x

[59] Mackerras DE, Hutton SI, Anderson PR (2004) Haematocrit levels and anaemia in Australian children aged 1–4 years. Asia Pac J Clin Nutr 13(4):330–335 15563436

[60] Rebuli MA, Williams G, James-Martin G, Hendrie GA (2020) Food group intake at self-reported eating occasions across the day: secondary analysis of the Australian National Nutrition Survey 2011–2012. Public Health Nutr 23(17):3067–3080 32690125 10.1017/S1368980020001585 PMC10200412

[61] Cole TJ, Bellizzi MC, Flegal KM, Dietz WH (2000) Establishing a standard definition for child overweight and obesity worldwide: international survey. BMJ (Clinical Res ed) 320(7244):1240–1243. 10.1136/bmj.320.7244.1240 10.1136/bmj.320.7244.1240 PMC27365 10797032

[62] Cole TJ, Flegal KM, Nicholls M, Jackson AA (2007) Body mass index cut offs to define thinness in children and adolescents: international survey. BMJ: Br Med J 335(7612):194–197 17591624 10.1136/bmj.39238.399444.55 PMC1934447

[63] Harttig U, Haubrock J, Knuppel S, Boeing H (2011) The MSM program: web-based statistics package for estimating usual dietary intake using the multiple source method. Eur J Clin Nutr S1:87 10.1038/ejcn.2011.92 21731011

