# Supplementary material for: Bioavailability of Australian pre-schooler iron intakes at specific eating occasions is low
Source: Eur J Nutr. 2024 Jun 14;63(7):2587–98. doi: 10.1007/s00394-024-03441-8 (PMC11490464; doi:10.1007/s00394-024-03441-8)

Bioavailability of Australian pre-schooler iron intakes at specific eating occasions is low

European Journal of Nutrition *Online Supporting Material*

Linda A. Atkins, Sarah A. McNaughton, Alison C. Spence, Lenore J. Evans, Rebecca M. Leech, Ewa A. Szymlek-Gay
Deakin University, Burwood, Australia, Institute for Physical Activity and Nutrition (IPAN), School of Exercise and Nutrition Sciences
Correspondence: ewa.szymlekgay@deakin.edu.au

Online Supplementary Fig. 1 Participant flow chart

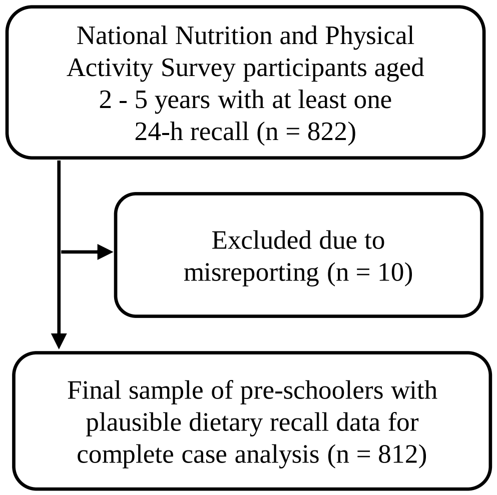

Supplement: Supplementary file 1 — Supplementary Material 1 [file 394_2024_3441_MOESM1_ESM.pdf]
