# Supplementary material for: Bioavailability of Australian pre-schooler iron intakes at specific eating occasions is low
Source: Eur J Nutr. 2024 Jun 14;63(7):2587–98. doi: 10.1007/s00394-024-03441-8 (PMC11490464; doi:10.1007/s00394-024-03441-8)
Supplement: Supplementary file 2 — Supplementary Material 2 [file 394_2024_3441_MOESM2_ESM.pdf]

**Bioavailability of Australian pre-schooler iron intakes at specific eating occasions is low**
***European Journal of Nutrition*** *Online Supporting Material*
Linda A. Atkins, Sarah A. McNaughton, Alison C. Spence, Lenore J. Evans, Rebecca M. Leech, Ewa A. Szymlek-Gay
Deakin University, Burwood, Australia, Institute for Physical Activity and Nutrition (IPAN), School of Exercise and Nutrition Sciences
Correspondence: ewa.szymlekgay@deakin.edu.au

**Online Supplementary Table 1 Algorithms applied to estimate iron bioavailability of the diets of Australian pre-schoolers**

| Author, Year<br>Basis | Study population | Dietary data required for model in addition to heme/non-heme iron |
|---|---|---|
| Monsen & Balintfy, 1982 [20]<br>Modification of Monsen et al., 1978 algorithm. Revised the way absorption of non-heme iron was calculated based on a logarithmic relationship with enhancing factors, although absorption continued to range between 3 and 8%. | Adults<br>USA | Meat/Fish/Poultry<br>Vitamin C |
| Murphy et al., 1992 [22]<br>Developed algorithm for toddler diets high in tea. Based on algorithm of Monsen et al., 1978, but translated into nutrients/4.18 MJ and assumed 1/3 adult meal = 1 toddler meal to derive dietary intakes. | Toddlers<br>Egypt, Kenya & Mexico | Meat/Fish/Poultry<br>Vitamin C<br>Tea (polyphenol) |
| Tseng et al., 1997 [23]<br>Non-heme iron availability was adjusted for Meat/Fish/Poultry and vitamin C based on Monsen et al., 1978, and Monsen & Balintfy, 1982 and then further adjusted for presence of phytates and tea separately. | Women & children<br>Russia | Meat/Fish/Poultry<br>Vitamin C<br>Tea (polyphenol)<br>Phytate |
| Reddy et al., 2000 [25]<br>Based algorithm on results of regression analysis of test meals consumed by adults, which found only content of Meat/Fish/Poultry, phytate and ascorbic acid were useful in estimating non-heme iron absorption. Adjusted iron absorption of subjects to correspond to serum ferritin concentration of 30 μg/L. | Adults<br>USA | Meat/Fish/Poultry<br>Vitamin C<br>Phytate |
| Bhargava et al., 2001 [26]<br>Based on approach by Monsen et al., 1978, Monsen & Balintfy, 1982 and Tseng et al., 1997, with further adjustments for phytates. | Women<br>Bangladesh | Meat/Fish/Poultry<br>Iron from Meat/Fish/Poultry<br>Vitamin C<br>Phytate |

Linda A. Atkins, Sarah A. McNaughton, Alison C. Spence, Lenore J. Evans, Rebecca M. Leech, Ewa A. Szymlek-Gay
Deakin University, Burwood, Australia, Institute for Physical Activity and Nutrition (IPAN), School of Exercise and Nutrition Sciences
Correspondence: ewa.szymlekgay@deakin.edu.au

**Online Supplementary Table 2 Estimated bioavailability of dietary iron consumed across the day and at specific EOs of Day 2 by 2-3-y-olds from the 2011–12 Australian National Nutrition and Physical Activity Survey shown as the proportion of consumed iron that is bioavailable (median %) and amount that is absorbable (median (25th, 75th percentile) mg)**

| | Median observed iron intake | Median bioavailable iron | | | | | | | | | | Range | |
|---|---|---|---|---|---|---|---|---|---|---|---|---|---|
| | | Monsen & Balintfy 1982 [20] | | Murphy et al.1992 [22] | | Tseng et al. 1997 [23] | | Reddy et al. 2000 [25] | | Bhargava et al. 2001 [26] | | | |
| | mg (25th, 75th percentile) | % | mg (25th, 75th percentile) | % | mg (25th, 75th percentile) | % | mg (25th, 75th percentile) | % | mg (25th, 75th percentile) | % | mg (25th, 75th percentile) | % | mg |
| Median daily intake and bioavailability of iron consumed by children (n = 293) | 6.56 (4.53, 9.10) | **5.4** | 0.37 (0.25, 0.57) | **10.2** | 0.68 (0.46, 0.99) | **3.2** | 0.20 (0.12, 0.31) | **8.1** | 0.51 (0.34, 0.78) | **2.9** | 0.19 (0.11, 0.27) | **2.9 - 10.2** | 0.19 - 0.68 |
| Breakfast (n = 289) | 1.58 (0.88, 3.69) | **3.1** | 0.06 (0.03, 0.14) | **5.0** | 0.11 (0.05, 0.24) | **1.2** | 0.03 (0.01, 0.05) | **5.6** | 0.08 (0.04, 0.18) | **1.2** | 0.03 (0.01, 0.05) | **1.2 - 5.6** | 0.03 - 0.11 |
| Lunch (n = 270) | 1.22 (0.77, 1.74) | **4.9** | 0.06 (0.03, 0.11) | **15.0** | 0.12 (0.06, 0.22) | **2.3** | 0.02 (0.01, 0.06) | **6.9** | 0.07 (0.04, 0.13) | **2.2** | 0.02 (0.01, 0.05) | **2.2 - 15.0** | 0.02 - 0.12 |
| Dinner (n = 285) | 1.50 (0.91, 2.38) | **9.1** | 0.14 (0.07, 0.23) | **16.5** | 0.23 (0.12, 0.39) | **5.9** | 0.08 (0.04, 0.15) | **11.9** | 0.17 (0.08, 0.34) | **4.9** | 0.07 (0.03, 0.13) | **4.9 - 16.5** | 0.07 - 0.23 |
| Early snacks (n = 243) | 0.45 (0.23, 0.96) | **3.5** | 0.02 (0.01, 0.04) | **5.0** | 0.04 (0.02, 0.08) | **2.1** | 0.01 (0.00, 0.02) | **7.0** | 0.03 (0.02, 0.06) | **2.1** | 0.01 (0.00, 0.02) | **2.1 - 7.0** | 0.01 - 0.04 |
| Late snacks (n = 257) | 0.56 (0.29, 1.47) | **3.4** | 0.02 (0.01, 0.06) | **5.0** | 0.04 (0.02, 0.11) | **2.2** | 0.01 (0.01, 0.03) | **7.1** | 0.04 (0.02, 0.07) | **2.2** | 0.01 (0.01, 0.03) | **2.2 - 7.1** | 0.01 - 0.04 |

EO: eating occasion
Percentages are bold font for readability. Early snacks are snacks consumed after midnight and before midday. Late snacks are snacks consumed between midday and midnight.

Linda A. Atkins, Sarah A. McNaughton, Alison C. Spence, Lenore J. Evans, Rebecca M. Leech, Ewa A. Szymlek-Gay
Deakin University, Burwood, Australia, Institute for Physical Activity and Nutrition (IPAN), School of Exercise and Nutrition Sciences
Correspondence: ewa.szymlekgay@deakin.edu.au

**Online Supplementary Table 3 Estimated bioavailability of dietary iron consumed across the day and at specific EOs of Day 2 by 4-5-y-olds from the 2011–12 Australian National Nutrition and Physical Activity Survey shown as the proportion of consumed iron that is bioavailable (median %) and amount that is absorbable (median (25th, 75th percentile) mg)**

| | Median observed iron intake | Median bioavailable iron | | | | | | | | | | Range | |
|---|---|---|---|---|---|---|---|---|---|---|---|---|---|
| | | Monsen & Balintfy 1982 [20] | | Murphy et al.1992 [22] | | Tseng et al. 1997 [23] | | Reddy et al. 2000 [25] | | Bhargava et al. 2001 [26] | | | |
| | mg (25th, 75th percentile) | % | mg (25th, 75th percentile) | % | mg (25th, 75th percentile) | % | mg (25th, 75th percentile) | % | mg (25th, 75th percentile) | % | mg (25th, 75th percentile) | % | mg |
| Median daily intake and bioavailability of iron consumed by children (n = 205) | 7.31 (5.26, 9.60) | **5.4** | 0.40 (0.29, 0.58) | **10.2** | 0.71 (0.52, 1.01) | **3.0** | 0.20 (0.13, 0.30) | **7.7** | 0.51 (0.36, 0.78) | **2.7** | 0.17 (0.12, 0.26) | **2.7 - 10.2** | 0.17 - 0.71 |
| Breakfast (n = 201) | 2.05 (1.11, 3.92) | **3.2** | 0.08 (0.04, 0.15) | **5.0** | 0.15 (0.07, 0.26) | **1.2** | 0.03 (0.01, 0.05) | **4.8** | 0.09 (0.05, 0.18) | **1.2** | 0.03 (0.01, 0.05) | **1.2 - 5.0** | 0.03 - 0.15 |
| Lunch (n = 197) | 1.42 (1.04, 1.92) | **4.6** | 0.06 (0.04, 0.12) | **10.4** | 0.11 (0.06, 0.24) | **1.9** | 0.02 (0.01, 0.05) | **5.9** | 0.07 (0.04, 0.13) | **1.7** | 0.02 (0.01, 0.05) | **1.7 - 10.4** | 0.02 - 0.11 |
| Dinner (n = 200) | 1.85 (1.04, 2.84) | **9.2** | 0.15 (0.08, 0.25) | **16.4** | 0.26 (0.15, 0.40) | **5.6** | 0.09 (0.04, 0.15) | **12.1** | 0.19 (0.08, 0.36) | **4.6** | 0.07 (0.03, 0.12) | **4.6 - 16.4** | 0.07 - 0.26 |
| Early snacks (n = 155) | 0.47 (0.24, 1.14) | **3.5** | 0.02 (0.01, 0.05) | **10.0** | 0.04 (0.02, 0.09) | **2.1** | 0.01 (0.00, 0.02) | **7.0** | 0.03 (0.02, 0.07) | **2.1** | 0.01 (0.00, 0.02) | **2.1 - 10.0** | 0.01 - 0.04 |
| Late snacks (n = 187) | 0.57 (0.27, 1.10) | **3.5** | 0.02 (0.01, 0.05) | **5.0** | 0.04 (0.02, 0.09) | **1.9** | 0.01 (0.00, 0.02) | **6.7** | 0.03 (0.02, 0.06) | **1.9** | 0.01 (0.00, 0.02) | **1.9 - 6.7** | 0.01 - 0.04 |

EO: eating occasion
Percentages are bold font for readability. Early snacks are snacks consumed after midnight and before midday. Late snacks are snacks consumed between midday and midnight.
